# Prophage-encoded virulence factor, Gp05, contributes to endothelial cell dysfunction and immune evasion to promote persistent methicillin-resistant *Staphylococcus aureus* endovascular infections

**DOI:** 10.1128/mbio.01685-25

**Published:** 2025-09-08

**Authors:** Yi Li, Adhar C. Manna, Sarah Ibrahim, Richard A. Proctor, Ambrose L. Cheung, Yan Q. Xiong

**Affiliations:** 1 The Lundquist Institute for Biomedical Innovation at Harbor-UCLA Medical Center, Torrance, California, USA; 2 Department of Microbiology & Immunology, Geisel School of Medicine at Dartmouth, Hanover, New Hampshire, USA; 3 University of Wisconsin School of Medicine and Public Health 5232, Madison, Wisconsin, USA; 4 David Geffen School of Medicine at UCLA 12222 , Los Angeles, California, USA; The University of Texas Health Science Center at Houston, Houston, Texas, USA

**Keywords:** MRSA, phage-encoded virulence factor, endothelial cell, immune evasion, endovascular infections

## Abstract

Methicillin-resistant *Staphylococcus aureus* (MRSA) is a leading cause of endovascular infections, where interactions with endothelial cells play a critical role in pathogenesis. Gp05, a prophage-encoded protein, has previously been implicated in promoting antibiotic persistence by modulating MRSA cellular physiology and evading neutrophil-mediated killing. In this study, we investigated the role of Gp05 in MRSA–endothelial cell interactions, focusing on its impact on bacterial adhesion, invasion, cytotoxicity, and the host inflammatory response. Using an isogenic MRSA strain set—including a clinical persistent bacteremia isolate (PB 300-169), a *gp05* deletion mutant, and a *gp05*-complemented strain—we found that deletion of *gp05* significantly impaired MRSA invasion, intracellular survival, and damage toward endothelial cells. Notably, endothelial cells infected with the *gp05* deletion mutant induced a heightened endothelial inflammatory response, characterized by increased production of cytokines (IL-1β, TNF-α, IFN-γ, and CCL2/MCAF) and upregulated expression of inflammatory- and adhesion-associated molecules (VEGF, VCAM-1, TLR2, and TLR6). Furthermore, treatment of endothelial cells with purified Gp05 protein alone was sufficient to suppress endothelial inflammatory signaling and induce cytotoxic effects, suggesting that Gp05 exerts both intracellular and extracellular functions: intracellularly, by attenuating host immune activation to promote bacterial survival, and extracellularly, by directly disrupting endothelial barrier integrity and dampening immune recognition. In conclusion, Gp05 is a key virulence factor that contributes to MRSA persistence in endovascular infections by promoting endothelial cell dysfunction, suppressing host immune response, and enhancing bacterial survival. These findings highlight Gp05 as a potential therapeutic agent for disrupting MRSA pathogenesis in vascular tissues.

**IMPORTANCE:**

This study reveals the critical role of Gp05, a prophage-encoded protein, in promoting antibiotic persistence during MRSA endovascular infections by modulating endothelial cell responses. By demonstrating that Gp05 enhances *S. aureus* endothelial cell invasion, intracellular survival, and cytotoxicity, while simultaneously suppressing host immune signaling, the research highlights Gp05 as a dual-function factor with both intracellular and extracellular effects on MRSA–host endothelial cell interactions. The identification of Gp05’s capacity to disrupt endothelial cells and dampen host immune system advances our understanding of the mechanism of MRSA persistence. Given the clinical challenges of treating persistent MRSA infections, especially in endovascular contexts, these findings position Gp05 as a compelling target for novel therapeutic strategies.

## INTRODUCTION

Methicillin-resistant *Staphylococcus aureus* (MRSA) is a major cause of endovascular infections, including bacteremia and infective endocarditis (IE) (1, 2). Despite treatment with first-line anti-MRSA antibiotics such as vancomycin (VAN) and daptomycin (DAP), clinical failure rates remain alarmingly high, with mortality approaching 30%, even in cases caused by MRSA strains deemed susceptible by Clinical Laboratory Standards Institute (CLSI) guidelines (3, 4). One particularly challenging manifestation is persistent bacteremia (PB), defined as positive blood cultures for five or more days despite appropriate antibiotic therapy, which occurs in 15–30% of MRSA endovascular infections and reflects a distinct form of antibiotic failure not fully explained by traditional resistance mechanisms (4, 5).

A key contributor to this failure is *S. aureus*’s ability to evade host immune responses. Through mechanisms that inhibit immune activation, MRSA can adhere to host cells and extracellular matrix ligands, facilitating colonization and impeding bacterial clearance (6). Increasing evidence highlights the critical role of endothelial cells in both the pathogenesis and persistence of MRSA during endovascular infections (7, 8). MRSA can adhere to, invade, and persist within endothelial cells, thereby escaping immune surveillance and reducing susceptibility to antibiotic treatment (9–11). Thus, elucidating the molecular mechanisms governing these host-pathogen interactions is essential for the development of novel therapeutic strategies aimed at improving treatment outcomes in MRSA-associated endovascular infections.

Our recent study identified Gp05, a prophage-encoded protein, as a novel virulence factor that promotes antibiotic persistence in an experimental MRSA endocarditis model (12). Gp05 enhances MRSA survival during VAN treatment by modulating multiple pathogenic pathways, including GraSR two-component regulatory system, tricarboxylic acid (TCA) cycle activity, and the stringent response (12, 13). These adaptations contribute to enhanced persistence and immune evasion. However, the specific role of Gp05 in host-pathogen interactions, particularly its effect on MRSA–endothelial cell dynamics, remains uncharacterized.

We hypothesize that Gp05 facilitates MRSA immune evasion and persistence through direct modulation of host cells during infection. To test this, we investigated the role of Gp05 in MRSA–endothelial cell interactions using an isogenic strain set, including wild-type PB MRSA 300-169, its *gp05* chromosomal deletion mutant, and *gp05*-complemented strains. In addition, we assessed the impact of exogenously applied synthesized and purified recombinant Gp05 on endothelial cells to evaluate its potential role as a secreted effector protein. Our findings provide new insights into the dual function of Gp05, as both a facilitator of intracellular persistence and an extracellular modulator of host immune signaling, thereby establishing its role in MRSA immune evasion and endothelial dysfunction. These results lay the groundwork for future therapeutic approaches targeting persistent MRSA endovascular infections.

## RESULTS

### Gp05 promotes MRSA endothelial cell invasion and damage

**Fig 1:**
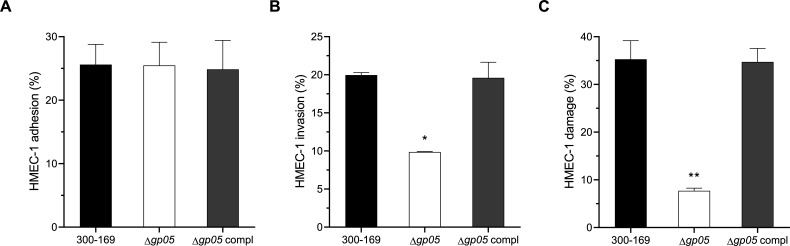
Adhesion (**A**), invasion (**B**), and damage (**C**) of HMEC-1 cells by the PB MRSA 300-169 strain set. *P < 0.05, **P < 0.01 vs. the WT and *gp05*-complemented strains.

Since Gp05 associates with persistent MRSA endocarditis, we hypothesized that Gp05 contributes to increased endothelial cell adhesion, invasion, and damage. The results demonstrated that the *gp05* deletion mutant exhibited significantly reduced HMEC-1 cell invasion and damage, compared to the wild-type and *gp05*-complemented strains (Fig. 1). However, the deletion of *gp05* did not significantly affect the adhesion to HMEC-1 cells, as the three study strains displayed similar adhesion capabilities (Fig. 1A). These results suggest that while Gp05 does not directly influence endothelial cell adhesion, it plays a critical role in assisting MRSA’s interactions with endothelial cells, enhancing its invasive capacity and cytotoxic effects.

### Gp05 antagonizes VAN reduction MRSA adhesion, invasion, and damage to HMEC-1 cells

**Fig 2:**
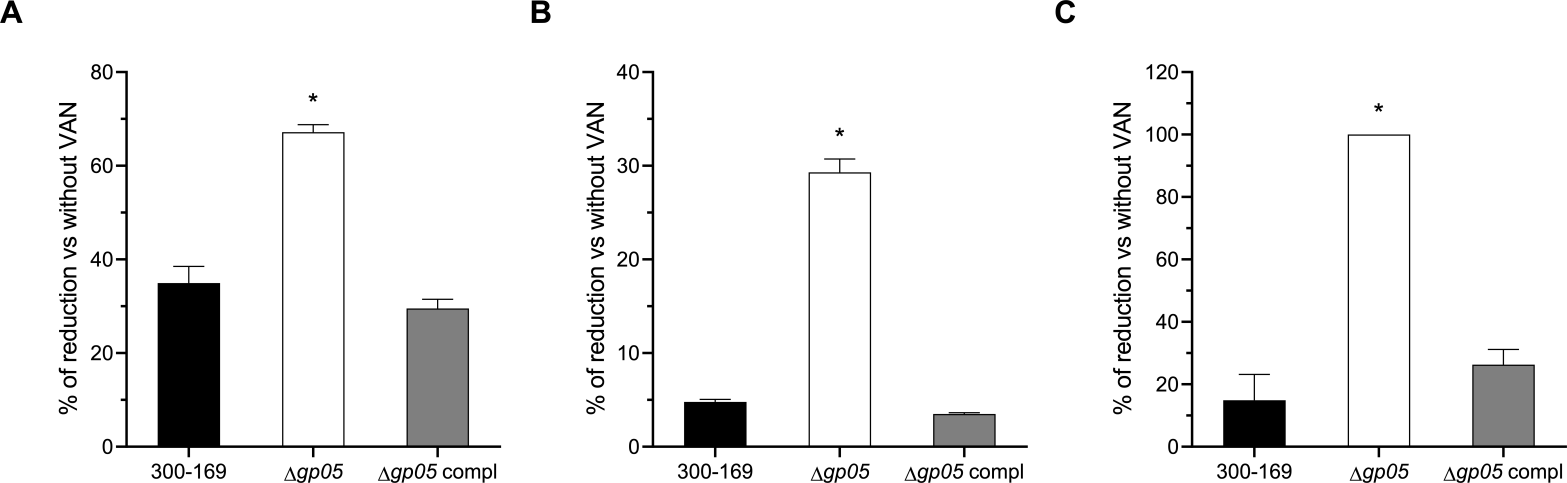
Reduction in HMEC-1 adhesion (**A**), invasion (**B**), and damage (**C**) by the PB MRSA 300-169 strain set upon supplementation with 1/2 MIC of VAN compared to results without VAN. *P < 0.01 vs. the WT and *gp05*-complemented strains.

Gp05-carrying MRSA isolates are less responsive to VAN treatment in a rabbit IE model, implying Gp05’s contribution to VAN persistence in MRSA (12). This finding led us to hypothesize that VAN plays a critical role in Gp05-related MRSA–endothelial cell interactions. To test this, we assessed the HMEC-1 adhesion, invasion, and damage caused by the study strains in the presence of 1/2 MIC of VAN. The results revealed that VAN reduced HMEC-1 cell adhesion, invasion, and damage across all study strains. Importantly, the *gp05* deletion mutant displayed a significantly greater reduction in all these assays compared to the wild-type and *gp05*-complemented strains (Fig. 2). These findings suggest that VAN affects MRSA infection by disrupting MRSA–endothelial cell interactions and that Gp05 contributes to MRSA persistence by weakening VAN’s effects.

### Gp05 enhances MRSA intracellular survival in HMEC-1 cells

**Fig 3:**
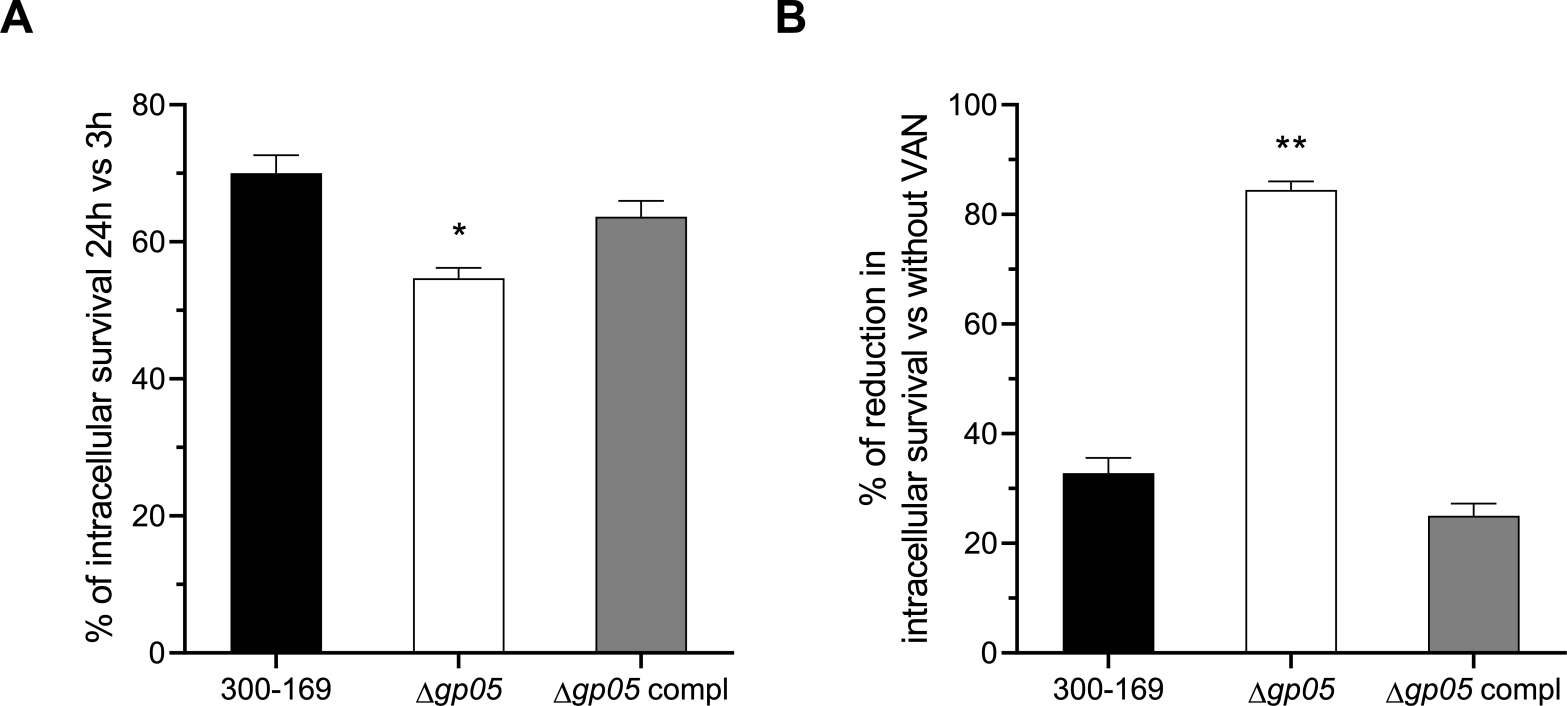
Intracellular survival of the PB MRSA 300-169 strain set in HMEC-1 cells at 24 h compared to 3 h (**A**), and reduction in intracellular survival upon supplementation with 1/2 MIC of VAN, compared to results without VAN (**B**). *P < 0.01, **P < 0.001 vs. the WT and *gp05*-complemented strains.

To investigate the role of Gp05 in MRSA endothelial cell intracellular survival, we tested the study strains and found that the *gp05* deletion mutant exhibited significantly lower intracellular survival compared to the wild-type and *gp05*-complemented strains (Fig. 3A). When treated with 1/2 MIC of VAN, the intracellular survival rate of the *gp05* deletion mutant strain was reduced by over 80%, whereas the wild-type and *gp05*-complemented strains showed only moderate reductions in intracellular survival under the same conditions (Fig. 3B), which might be the consequence of reduced MRSA adhesion and invasion caused by VAN. These findings indicate that Gp05 enhances MRSA’s intracellular survival within HMEC-1 cells, potentially by modulating bacterial responses or endothelial cell interactions to reduce susceptibility to treatment.

### Exogenous Gp05 enhances HMEC-1 cell invasion and damage of MRSA

**Fig 4:**
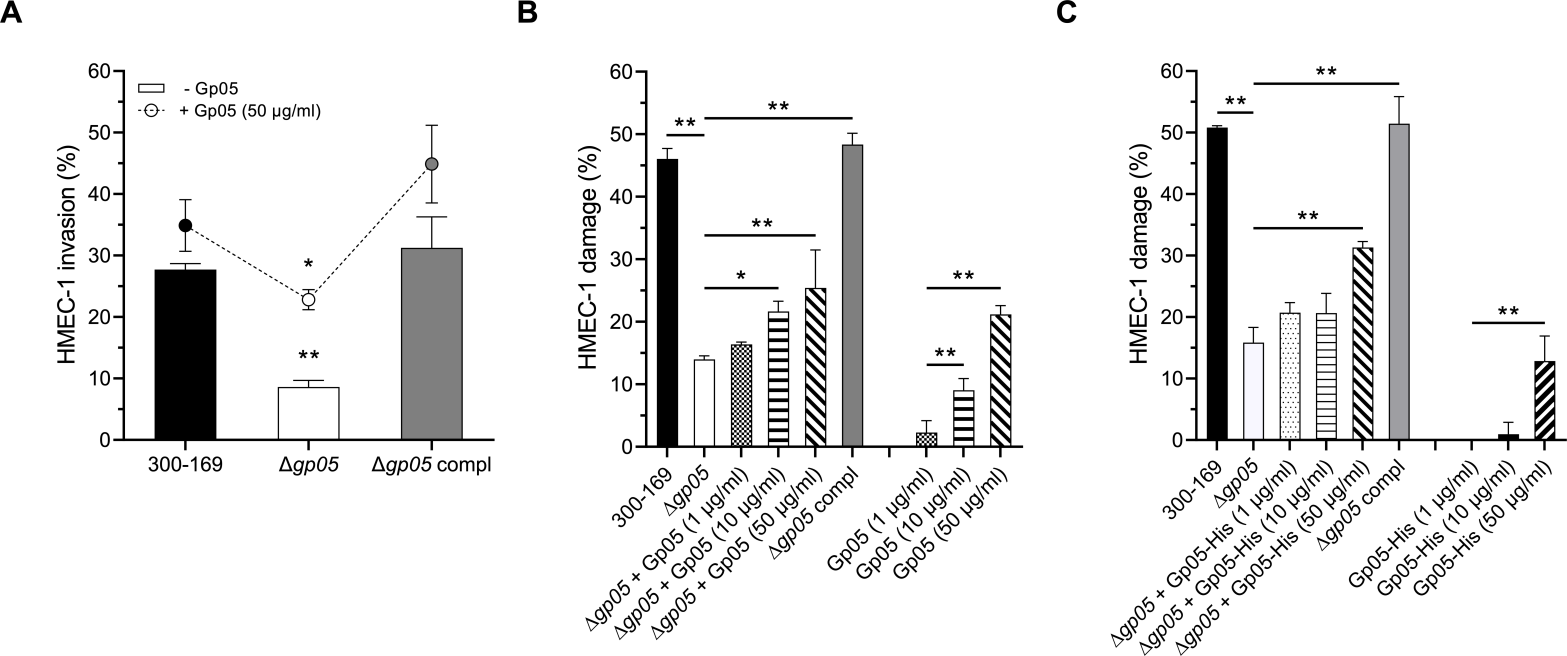
HMEC-1 invasion (**A**) and damage (**B**) caused by PB MRSA 300-169 strain set with and without supplementation of synthesized Gp05 and HMEC-1 damage induced by synthesized Gp05 alone. The HMEC-1 damage assays were repeated using the recombinant Gp05-His (**C**). *P < 0.05, **P < 0.001 vs. the WT and *gp05*-complemented strains.

Gp05 is a secreted protein (12), suggesting it may interact not only with neighboring *S. aureus* cells but also with host cells in the extracellular environment during infection. To evaluate the extrinsic effects of Gp05 on both MRSA and host endothelial cells, we supplemented HMEC-1 assays with synthesized Gp05. At a high concentration (50 µg/mL), synthesized Gp05 significantly enhanced MRSA invasion into HMEC-1 cells across all tested strains, with HMEC-1 cell invasion by the *gp05* deletion mutant strain increasing by over 100% (Fig. 4A). Synthesized Gp05 also markedly increased HMEC-1 cell damage caused by the *gp05* deletion mutant strain in a concentration-dependent manner (Fig. 4B). Furthermore, synthesized Gp05 alone, without MRSA exposure, induced direct, concentration-dependent lytic of HMEC-1 cells (Fig. 4B). Importantly, supplementation of the *gp05* deletion mutant with recombinant Gp05-His (produced C-terminal His_6_-tag fusion in *E. coli*) restored its capacity to damage HMEC-1 cells (Fig. 4C), and Gp05-His alone induced cytotoxicity independent of MRSA infection (Fig. 4C). These findings underscore the dual role of Gp05: it promotes MRSA invasion and endothelial cell damage during infection and also acts extracellularly as a cytotoxin, directly contributing to host cell damage independently of bacterial presence.

### Gp05 represses HMEC-1 cells' inflammatory response

**Fig 5:**
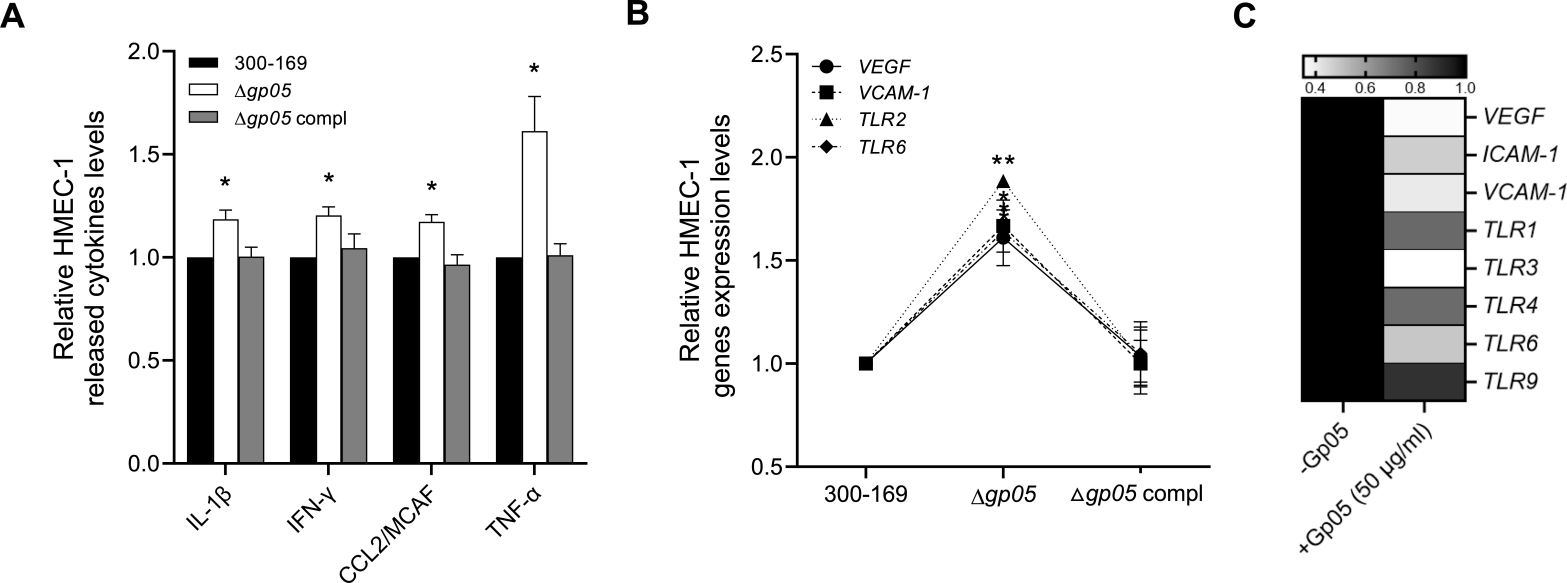
Cytokine levels (**A**) and gene expression levels (**B**) of HMEC-1 cells infected by the 300-169 strain set. Gene expression levels in HMEC-1 treated with synthesized Gp05 (**C**). Black color indicates a relative expression level of one; darker colors represent higher expression levels. *P < 0.05, **P < 0.01 vs. the WT and *gp05*-complemented strains.

We have demonstrated that Gp05 contributes to endothelial cell dysfunction and persistent MRSA endovascular infections (12). Here, we investigated how Gp05 influences endothelial inflammatory responses. The results showed that HMEC-1 cells infected with the *gp05* deletion mutant strain exhibited significantly higher production of IL-1β, IFN-γ, MCAF, and TNF-α compared to those infected with the wild-type and *gp05*-complemented strains (Fig. 5A), suggesting that Gp05 suppresses endothelial cell inflammatory responses during MRSA infection, potentially aiding immune evasion. Consistent with this finding, HMEC-1 cells infected by the *gp05* deletion mutant strain also showed elevated transcription levels of genes encoding vascular endothelial growth factor (VEGF), vascular cell adhesion molecule-1 (VCAM-1), toll-like receptor 2 (TLR2), and TLR6 compared to those infected by wild-type and *gp05*-complemented strains (Fig. 5B), again suggesting that Gp05 presence might result in decreased signaling for recruiting inflammatory cells.

To further assess the impact of Gp05 on endothelial inflammatory response, we examined the cytokine levels and transcription of the genes encoding immune-related proteins of HMEC-1 cells treated with synthesized Gp05. Significant reductions in the transcription of genes encoding VEGF, intercellular adhesion molecule-1 (ICAM-1), VCAM-1, and multiple TLRs, including TLR1, TLR3, TLR4, TLR6, and TLR9, were observed with Gp05 exposure compared to untreated controls (Fig. 5C). However, the cytokine levels of HMEC-1 cells were not significantly affected by synthesized Gp05 compared to untreated controls (data not shown).

## DISCUSSION

Gp05 is critical for VAN persistence in MRSA endovascular infection by mediating both genotypic and phenotypic traits (12, 13). We explored the effects of Gp05 on MRSA cellular processes in previous studies, but its interaction with the host cells remained undiscovered. The present study bridges this knowledge gap and provides novel insights into Gp05-driven endothelial cell responses. The findings demonstrate that Gp05 exerts its effects on endothelial cells both intracellularly and extracellularly, influencing cellular damage, inflammatory signaling pathways, and immune receptor expression. These multifaceted effects of Gp05 not only enhance MRSA’s ability to adhere to, invade, and survive within HMEC-1 cells but also suppress endothelial cells’ inflammatory responses. This highlights Gp05’s dual role in promoting bacterial persistence and modulating host immune responses, thereby contributing to the severity and chronicity of MRSA endovascular infections.

In this study, we uncovered the ability of Gp05 to facilitate MRSA invasion, intracellular survival, and damage to endothelial cells, while also revealing its differential impact on bacterial adhesion to endothelial cells under antibiotic pressure. The *gp05* deletion mutant strain demonstrated significantly reduced HMEC-1 invasion, intracellular survival, and cellular damage compared to the wild-type and *gp05*-complemented strains. This suggests that Gp05 is required for MRSA to successfully invade and persist within endothelial cells while also magnifying host cell injury. These findings align with previous observations of Gp05’s role in promoting bacterial persistence and survival under host stress conditions (12), underscoring its functional significance in the pathogenesis of MRSA endovascular infections. Interestingly, supplementation with sub-MIC VAN amplified the observed differences among the strains and further suppressed the ability of the *gp05* mutant strain to invade, damage, and survive intracellularly within HMEC-1 cells, while the wild-type and *gp05*-complemented strains retained significantly higher levels of these processes. The remarkable sensitivity of the *gp05* deletion mutant strain to VAN highlights the interplay between Gp05-mediated persistence and antibiotic susceptibility in the context of host-pathogen interactions. An intriguing observation in this study was the effect of Gp05 on bacterial adhesion to HMEC-1 cells. Endothelial cell adhesion is an important step in *S. aureus* pathogenesis and is closely associated with persistent MRSA endovascular infections (8, 10, 14). In endovascular infections, adhesion to endothelial cells allows *S. aureus* to establish a localized infection and evade clearance by the host immune system (14). Previous studies have demonstrated that PB MRSA strains exhibit greater adherence to endothelial cells (8, 15), along with significantly higher invasion and damage to endothelial cells compared to RB MRSA strains (7, 8). Under normal conditions (without antibiotic exposure), the *gp05* deletion mutant strain exhibited similar levels of adhesion to HMEC-1 cells compared to the wild-type and *gp05*-complemented strains, indicating that Gp05 does not play a major role in the basic adhesion processes. However, the addition of sub-MIC VAN significantly reduced the HMEC-1 adhesion of the *gp05* deletion mutant strain while having a lesser effect on the wild-type and *gp05*-complemented strains. This suggested that Gp05 may function in maintaining the ability of MRSA to adhere to endothelial cells under antibiotic pressure, resulting in subsequent pathogen persistence during treatment. These findings at least partially reinforce Gp05’s contribution in MRSA’s tolerance to VAN treatment during endothelial infection.

Endothelial cells are key mediators of inflammatory responses, releasing cytokines such as IL-1β, IFN-γ, TNF-α, and chemokines like CCL2/MCAF (16, 17). These molecules regulate immune cell recruitment, modulate endothelial barrier integrity, and promote vascular inflammation. One of the key observations in this study was the increased production of pro-inflammatory cytokines (e.g., IL-1β, IFN-γ, and TNF-α) and chemokines (CCL2/MCAF), as well as the increased expression of genes encoding VEGF, VCAM-1, TLR2, and TLR6 in HMEC-1 cells infected by *gp05* deletion mutant strain. These results suggest that Gp05 plays a critical role in modulating endothelial inflammatory responses to support *S. aureus* persistence. IL-1β and TNF-α, key mediators of neutrophil recruitment and immune cell adhesion (18–21), were significantly higher in *gp05* deletion mutant-infected HMEC-1 cells, implying that Gp05 suppresses these innate immune responses to facilitate immune evasion and bacterial survival. Similarly, increased IFN-γ and CCL2/MCAF production in the absence of Gp05 highlights its role in limiting immune activation, as both molecules are essential for promoting monocyte recruitment (22–24). Although the differences observed in the four cytokines were relatively small, they were statistically significant and consistent with the suppressive effects of Gp05 on endothelial immune signaling. The multiplex ELISA kit also included four additional cytokines (IL-α, IL-6, IL-8, and GM-CSF), which showed no significant changes among the study strains, suggesting that Gp05 selectively modulates specific inflammatory pathways rather than broadly altering cytokine production. The upregulation of genes encoding VEGF and VCAM-1 in the *gp05* mutant infections further underscores Gp05’s regulatory role in endothelial activation and angiogenic responses. VEGF, a central regulator of angiogenesis and immune cell recruitment, stimulates the expression of adhesion molecules like ICAM-1 and VCAM-1 to promote leukocyte trafficking, while VCAM-1 is critical for immune cell migration and endothelial barrier regulation (25–27). The increased expression of these molecules in the absence of Gp05 suggests that it suppresses endothelial signaling pathways that support immune defense. Additionally, the elevated expression of TLR2 and TLR6 in *gp05* deletion mutant-infected HMEC-1 cells highlights the role of Gp05 in dampening pathogen recognition and inflammatory signaling. TLR2 and TLR6 are key components of the innate immune response, recognizing pathogen-associated molecular patterns (PAMPs) and triggering downstream inflammatory cascades through NF-κB activation (28, 29). By suppressing these pathways, Gp05 likely limits the immune system’s ability to detect and respond to *S. aureus* infection, facilitating bacterial persistence. These findings collectively reveal Gp05 as a regulator of endothelial cell inflammatory responses, balancing the activation of cytokine and adhesion molecule pathways to optimize bacterial survival and host immune evasion.

Additionally, we demonstrate that treatment of HMEC-1 cells with synthesized Gp05 led to a remarkable decrease in the expression of the genes encoding pro-inflammatory molecules, including VEGF, ICAM-1, VCAM-1, and various TLRs (e.g., TLR-1, TLR-3, TLR-4, TLR-6, and TLR-9). These results indicate that Gp05, independent of bacterial presence, directly engages host signaling pathways to restrain angiogenic and inflammatory responses. The downregulation of ICAM-1 and VCAM-1 suggests diminished endothelial activation and leukocyte adhesion, which are critical for immune cell recruitment (30). Moreover, the broad downregulation of TLRs highlights Gp05’s role in weakening innate immune pathways, potentially interfering with pathogen recognition and disrupting host inflammatory responses. Interestingly, synthesized Gp05 led to the downregulation of a wider range of pro-inflammatory molecules compared to the study strain (Fig. 5C vs. 5B). This effect may be attributed to the exceptionally high concentration of synthesized Gp05. On the other hand, TLR2 expression remained unaffected by synthesized Gp05, possibly due to the inherently low expression of TLR2 in HMEC-1 cells (31). Beyond its immunomodulatory effects, synthesized Gp05 directly caused significant HMEC-1 cell damage, demonstrating its cytotoxic capacity. This direct damage, combined with its ability to suppress pro-inflammatory signaling, underscores Gp05’s comprehensive contribution to vascular dysfunction during *S. aureus* infections. By simultaneously blocking immune activation and inducing endothelial cell cytotoxicity, Gp05 exacerbates endothelial dysfunction, fostering a microenvironment conducive to bacterial persistence, dissemination, and immune dysregulation.

In summary, the present research unveils the role of Gp05 in mediating the interaction between MRSA and endothelial cells. Gp05 enhances MRSA’s ability to invade and survive intracellularly within endothelial cells while modulating endothelial cell inflammatory signaling, contributing to immune evasion and bacterial persistence. Additionally, the extracellular effects of Gp05, including cytotoxicity and suppression of immune pathways, may further promote vascular damage and immune dysregulation, facilitating bacterial dissemination. Mechanistic studies to better understand how Gp05 induces these effects, such as identifying its receptor(s) on endothelial cells, are currently underway in our lab. These investigations may yield critical insights into the pathogenic role of Gp05 and uncover novel therapeutic targets for treating persistent MRSA endovascular infections.

## MATERIALS AND METHODS

### MRSA strains

To investigate the impact of Gp05 on host endothelial cell, we used a *gp05* deletion strain set derived from a clinical PB MRSA (300-169) strain, as previously constructed (12). All the strains exhibited the same VAN minimum inhibitory concentration (MIC) of 0.5 µg/mL. Unless otherwise specified, strains were routinely grown at 37°C in tryptic soy broth (TSB; Becton Dickinson and Company, Franklin Lakes, NJ, USA) or on tryptic soy agar (TSA) plates.

### Synthesis of Gp05 and purification of recombinant Gp05-His protein

Gp05 (accession: EUN40083; purity: 95.91%; molecular weight [MW]: 6391.93 Da) was chemically synthesized by Biomatik Corporation (Wilmington, DE, USA) using standard solid-phase methods. The purified peptide was further refined via reverse-phase high-performance liquid chromatography (HPLC) and analyzed by mass spectrometry (MS) to confirm its identity and purity. For all assays, the synthesized Gp05 was dissolved in MCDB131 medium (Sigma-Aldrich, St. Louis, MO, USA).

Cloning of the *gp05* gene for protein expression was performed by PCR amplification of 165 bp DNA fragment containing the open reading frame using chromosomal DNA from PB 300-169 and the primers 5′-AAG GAA AGT CAT ATG AAA ATA ACT AAT TGC AAA ATA AAA AAA-3′ and 5′-AAG GAA AGT CTC GAG ATT TAA CTT ATC GCC ATC TAT TTT TTG TGA -3′. PCR product was digested and ligated into the *NdeI* and *XhoI* sites of pET22b vector (Novagen, Madison, WI, USA) that contains a His_6_ tag sequence. Proper insertion of the *gp05* gene in the recombinant plasmid was confirmed by restriction digestion and by DNA sequencing. An authenticated recombinant plasmid was transformed into BL21pLysS *Escherichia coli* (*E. coli*) strain (Novagen) for protein expression. Protein expression was induced in broth culture with 1 mM isopropyl-ß-D-thiogalactopyranoside (IPTG) for 3 h. The cells were harvested and lysed by ultrasonication and ultracentrifuged for cell-free lysate. Gp05-His protein was purified on a His-tag purification column (Novagen) according to the manufacturer’s protocols. The purity of Gp05-His was dialyzed to remove salt and imidazole and confirmed by sodium dodecyl sulfate-polyacrylamide gel electrophoresis (SDS-PAGE) and protein staining with AcquaStain (Bulldog Bio, Portsmouth, NH, USA). The concentration of the purified protein was determined by the Bradford protein assay (Bio-Rad, Hercules, CA, USA), using bovine serum albumin (BSA) as the standard.

### Human endothelial cells

The human microvascular endothelial cell (HMEC-1) line was obtained from Kathryn Kellar at the Centers for Disease Control (CDC), USA, and maintained according to established protocols (32). Primary cells were derived from human dermal microvascular endothelial cells and immortalized via transfection with a pBR322-based plasmid containing the coding region for the simian virus 40 large T-antigen (32). HMEC-1 cells were routinely cultured in complete MCDB131 medium (Sigma-Aldrich, St. Louis, MO, USA), supplemented with 20% bovine calf serum, 2 mM glutamine, 100 IU/mL penicillin, and 100 mg/mL streptomycin, at 37°C in a 5% CO_2_ atmosphere (33, 34).

### MRSA adherence to HMEC-1 cells

Overnight cultures of MRSA (~5 × 10^3^ CFU/well) were added to HMEC-1 cells seeded in 24-well plates at a density of ~1 × 10^5^ HMEC-1 cells/well in full MCDB131 medium. The multiplicity of infection (MOI) was set to 5, as our preliminary experiments determined that the wild-type MRSA strain 300-169 induces approximately 50% HMEC-1 damage at this ratio. Following a 1-hour incubation, non-adherent MRSA cells were removed by washing the wells with Hank’s Balanced Salt Solution (HBSS, Thermo Fisher Scientific, Waltham, MA, USA). Ice-cold water was then added to lyse HMEC-1 cells and release bound MRSA. The number of adherent MRSA was quantified by plating serial dilutions on TSA plates (35). Bacterial adherence was expressed as the percentage (± SD) of MRSA bound to HMEC-1 cells relative to the initial inoculum (36).

### Endothelial cell damage

The impact of MRSA strains on endothelial cell integrity was assessed using a well-established 3-(4,5-dimethylthiazol-2-yl)-2,5-diphenyltetrazolium bromide (MTT) assay, as previously described (33, 34, 37). Briefly, overnight cultures of MRSA (5 × 10^5^ CFU/well) were co-incubated with HMEC-1 cells in 24-well plates at a density of approximately 1 × 10^5^ endothelial cells/well in complete MCDB131 medium, achieving a MOI of 5. Following a 3-hour infection period, extracellular MRSA cells were eliminated by treatment with lysostaphin (10 µg/mL) in complete MCDB131 medium, supplemented with 20% bovine calf serum, 2 mM glutamine, 100 IU/mL penicillin, and 100 mg/mL streptomycin (33, 34). After an additional 18-hour incubation at 37°C, MTT (5 mg/mL; Sigma-Aldrich, St. Louis, MO, USA) was added in Hank’s Balanced Salt Solution (HBSS, Thermo Fisher Scientific, Waltham, MA, USA) and incubated for 2 h. The reaction was then halted by replacing the medium with 0.04 M HCl in absolute isopropanol (Thermo Fisher Scientific, Waltham, MA, USA), ensuring complete cell lysis. Absorbance at 560 nm (A_560nm_) was measured using a Synergy 2 microplate reader (BioTek, Winooski, VT, USA). Uninfected HMEC-1 served as a negative control. Endothelial cell damage was calculated using the following formula: 1 − (A_560nm_ of test well/A_560nm_ of 0% − damage control well) as previously described (37).

### MRSA invasion of HMEC-1 cells

The ability of MRSA strains to invade endothelial cells was assessed using the lysostaphin protection assay, following a protocol similar to the HMEC-1 damage assay described above (7). Briefly, HMEC-1 cells were incubated with MRSA for 3 h to allow bacterial invasion. Subsequently, extracellular MRSA cells were selectively lysed by treatment with 10 µg/mL lysostaphin in full MCDB131 medium. To quantify internalized bacteria, HMEC-1 were lysed by exposure to ice-cold water, and the number of intracellular MRSA was determined through quantitative culturing, as previously described (35). The invasion rate was expressed as the percentage of internalized MRSA relative to the total bacterial population at the 3-hour time point, as previously described (7).

### MRSA intracellular survival within HMEC-1 cells

The intracellular survival rate of MRSA in HMEC-1 cells was determined using conditions similar to the HMEC-1 damage assay described above. Briefly, following a 3-hour invasion period, extracellular MRSA cells were removed by 10 µg/mL lysostaphin in full MCDB131 medium and then incubated for 24 h. The number of internalized MRSA and MRSA-infected HMEC-1 cells was determined as previously described (35). Intracellular survival was calculated based on the number of internalized MRSA per HMEC-1 cell at the 24-hour time point.

### Pro-inflammatory cytokine production

The levels of pro-inflammatory cytokines released by MRSA-infected HMEC-1 were quantified using a multiplex human cytokine ELISA kit (MyBioSource, San Diego, CA, USA). Briefly, culture supernatants were collected 3 h post-infection to assess cytokine concentrations according to the manufacturer’s instructions. The number of MRSA-infected HMEC-1 cells was determined as previously described (35). Cytokine levels were expressed as OD_450nm_ per million HMEC-1 cells and subsequently normalized to the cytokine levels of the wild-type MRSA-infected HMEC-1 cells.

### HMEC-1 RNA extraction and gene expression analysis

Total RNA was extracted from MRSA-infected HMEC-1 cells and subsequently reverse-transcribed into cDNA as previously described (15, 38). Briefly, following a 3-hour invasion period, the culture supernatant was removed, and TRI Reagent was added to the cell culture flask to lyse and detach infected HMEC-1 cells. The lysate was then centrifuged at 12,000 rpm for 10 min at 4°C, and the resulting supernatant containing HMEC-1 total RNA was collected for further purification using the RiboPure Kit (Invitrogen, Waltham, MA, USA) according to the manufacturer’s instructions. To eliminate residual genomic DNA, the RNA samples were treated with TURBO DNase Kit (Thermo Fisher, Waltham, MA, USA). Subsequently, 1 µg of DNase-treated RNA was reverse-transcribed into cDNA using the SuperScript III First-Strand Synthesis Kit (Invitrogen, Waltham, MA, USA) according to the manufacturer’s protocols. qRT-PCR was performed using an ABI Prism 7000 instrument (Applied Biosystems, Waltham, MA, USA) with SYBR^M^ Green PCR Master Kit (Applied Biosystems, Waltham, MA, USA). Primer sequences for the target HMEC-1 genes were obtained from previous studies (39, 40). *β-actin* was used as a housekeeping gene for normalization of transcript levels (41). Gene expression was quantified using the comparative threshold cycle (ΔΔCT) method (42).

### Statistical analysis

All experiments were performed in at least three independent biological replicates, with duplicates or triplicates per condition unless otherwise specified. Data are presented as mean ± standard deviation (SD). For comparisons involving more than two groups, one-way analysis of variance (ANOVA) was performed, followed by Tukey’s post hoc test to adjust for multiple comparisons. When only two groups were compared, an unpaired two-tailed Student’s *t*-test was used. *P* values < 0.05 were considered statistically significant. Statistical analyses were performed using GraphPad Prism 9 (GraphPad Software, San Diego, CA).
